# Diabetes and risk of Kaposi's sarcoma: effects of high glucose on reactivation and infection of Kaposi's sarcoma-associated herpesvirus

**DOI:** 10.18632/oncotarget.19685

**Published:** 2017-07-28

**Authors:** Pey-Jium Chang, Yao-Hsu Yang, Pau-Chung Chen, Lee-Wen Chen, Shie-Shan Wang, Ying-Ju Shih, Li-Yu Chen, Chi-Jen Chen, Chien-Hui Hung, Chun-Liang Lin

**Affiliations:** ^1^ Graduate Institute of Clinical Medical Sciences, College of Medicine, Chang-Gung University, Taoyuan, Taiwan; ^2^ Department of Nephrology, Chang-Gung Memorial Hospital, Chiayi, Taiwan; ^3^ Kidney and Diabetic Complications Research Team (KDCRT), Chang-Gung Memorial Hospital, Chiayi, Taiwan; ^4^ Department of Traditional Chinese Medicine, Chang-Gung Memorial Hospital, Chiayi, Taiwan; ^5^ Center of Excellence for Chang Gung Research Datalink, Chang-Gung Memorial Hospital, Chiayi, Taiwan; ^6^ Institute of Occupational Medicine and Industrial Hygiene, National Taiwan University College of Public Health, Taipei, Taiwan; ^7^ School of Traditional Chinese Medicine, College of Medicine, Chang Gung University, Taoyuan, Taiwan; ^8^ Department of Environmental and Occupational Medicine, National Taiwan University Hospital and National Taiwan University College of Medicine, Taipei, Taiwan; ^9^ Department of Respiratory Care, Chang-Gung University of Science and Technology, Chiayi, Taiwan; ^10^ Department of Pediatric Surgery, Chang-Gung Memorial Hospital, Chiayi, Taiwan

**Keywords:** Kaposi’s sarcoma, diabetes, KSHV, high glucose, lytic replication

## Abstract

Patients with diabetes are generally prone to pathogen infection and tumor progression. Here, we investigated the potential association between diabetes and Kaposi's sarcoma (KS), a tumor linked to infection with Kaposi's sarcoma-associated herpesvirus (KSHV). By using Taiwan's National Health Insurance Research Database, we found that diabetes is statistically associated with increased risk of KS in a case-control study. Since a high level of blood sugar is the hallmark of diabetes, we determined whether high glucose promotes both KSHV reactivation and infection, which are crucial for KS pathogenesis. Our results showed that high glucose significantly increases lytic reactivation of KSHV but not Epstein-Barr virus, another related human oncogenic gammaherpesvirus, in latently infected cells. Activation of the transcription factor AP1 by high glucose is critically required for the onset of KSHV lytic reactivation. We also demonstrated that high glucose enhances susceptibility of various target cells to KSHV infection. Particularly, in endothelial and epithelial cells, levels of specific cellular receptors for KSHV entry, including integrin α3β1 and xCT/CD98, are elevated under high glucose conditions, which correlate with the enhanced cell susceptibility to infection. Taken together, our studies implicate that the high-glucose microenvironment may be an important predisposing factor for KS development.

## INTRODUCTION

Kaposi's sarcoma (KS) is an endothelial cell-derived vascular tumor, which has four clinical forms including classic, African endemic, AIDS-related, and transplant-related KS [1]. All KS subtypes are linked to infection of Kaposi's sarcoma-associated herpesvirus (KSHV), or known as human herpesvirus 8 (HHV-8) [2]. Although latent infection with KSHV plays an essential role in viral persistence and tumorigenesis, numerous studies have shown that active lytic program of KSHV is also crucial for KS pathogenesis and strongly correlates with KS progression and severity [2–5].

Despite the fact that infection with KSHV is absolutely required for KS occurrence, primary viral infection alone may be not sufficient for tumorigenesis as certain people infected with KSHV do not manifest any symptoms [2]. Various genetic or nongenetic cofactors, independently or combined, were suggested to support the development of KS in infected subjects. Immune suppression caused by human immunodeficiency virus (HIV) co-infection or by other immune suppressants is considered as the major cofactor for KS pathogenesis [6–8]. Besides, in a population-based case-control study, Anderson et al. [9] reported that diabetes is a potential risk factor for classic KS development. Although accumulating evidence reveals that patients with diabetes are more susceptible to pathogen infection and are more likely to develop certain cancers [10, 11], the causal association between diabetes and KS still needs to be further demonstrated.

Diabetes is a metabolic disease diagnosed with high levels of sugar (glucose) in blood. Evidence has shown that high glucose can modulate multiple signaling pathways in various cell types [12–16]. Importantly, several high glucose-mediated signaling transducers or effectors, such as protein kinase C (PKC), reactive oxygen species, ERK1/2, JNK, p38, AP1, SP1 or Notch, appear to overlap with those involved in the induction of KSHV lytic reactivation [17–19]. Recently, studies from Ye et al. [20] showed that high glucose could reactivate KSHV lytic gene expression from latently infected cells. Despite such evidence, the detailed molecular mechanism underlying activation of viral lytic replication by high glucose is not yet fully understood. Furthermore, it remains unclear whether high glucose influences the susceptibility of target cells to KSHV infection.

In the report, we showed a positive relation between diabetes and the risk of KS development using the Taiwan National Health Insurance Research Database. Moreover, we discovered that high glucose increases not only KSHV reactivation in infected cells but also the susceptibility of target host cells to KSHV infection. The detailed molecular mechanisms of how high glucose modulates KSHV reactivation and KSHV infection were also characterized in the study. Our findings strongly suggest that high glucose is an important predisposing factor for KS development. Understanding the association between diabetes and KS may potentially provide further insights into diabetic complications and have impacts on KSHV pathogenesis.

## RESULTS

### Association of diabetes with increased risk of Kaposi's sarcoma

To study the association between diabetes and KS, data from the Taiwan National Health Insurance Research Database (NHIRD) between 1997 and 2008 were used in a case-control study. A total of 352 KS cases and 1, 408 matched controls (in a 1:4 ratio) were included in the analysis. The flow chart of the sampling procedures is shown in Supplementary Figure 1. The distribution of sex, age, income, levels of urbanization, and the proportions of comorbidities between subjects are shown in Table 1. Logistic regression analysis revealed that the adjusted odds ratio (OR) of diabetes in KS patients was 2.26 (95% CI 1.68 to 3.04, p<0.01) (Table 1), indicating that there is a significant relationship between diabetes and the risk of KS. After further adjusting for different covariates including end stage renal disease (ESRD), hypertension (HTN) and chronic hepatitis and cirrhosis CHC), the analysis still showed a similar estimate regarding the association of diabetes with the increased risk of KS based on these different models (Table 2). After stratified by subgroups according to sex, age and subjects with or without ESRD, HTN and CHC, the association between diabetes and increased risk of KS was further verified (Table 2).

**Table 1:** Demographic characteristics and adjusted odds ratio of Kaposi's sarcoma among Kaposi's sarcoma patients and control subjects

	KS cases (N = 352)		Controls (N = 1408)		p-value^*^	Adjusted^†^	
	No.	%	No.	%		OR	95%CI
Sex					1.00		
Female	63	17.9	252	17.9		1.00	
Male	289	82.1	1156	82.1		0.96	0.70 to 1.33
Age					0.89		
<65	155	44.0	614	43.6		1.00	
≥65	197	56.0	794	56.4		1.09	0.83 to 1.43
Median (IQR)	67.3 (56.6–76.1)		67.1 (56.3–76.1)				
Monthly income					1.00		
0	62	17.6	248	17.6		1.00	
1-15840	66	18.8	264	18.8		0.96	0.62 to 1.48
15841-25000	164	46.6	656	46.6		1.01	0.69 to 1.46
≥25001	60	17.1	240	17.1		1.05	0.67 to 1.64
Urbanization^‡^					1.00		
1	73	20.7	292	20.7		0.83	0.54 to 1.27
2	151	42.9	604	42.9		0.87	0.61 to 1.25
3	69	19.6	276	19.6		0.92	0.62 to 1.38
4	59	16.8	236	16.8		1.00	
Medical diseases^§^							
DM	87	24.7	193	13.7	<.01	2.26	1.68 to 3.04
HIV	31	8.8	0	0.0	<.01	>999	<.01 to >999
Transplant	1	0.3	0	0.0	0.05	>999	<.01 to >999
ESRD	7	2.0	3	0.2	<.01	—	
HTN	193	54.8	620	44.0	<.01	—	
CHC	116	33.0	323	22.9	<.01	—	

*Chi-square.

^†^Odds ratio (OR) adjusted for age, sex, income, urbanization, HIV and transplant.

^‡^Urbanization levels in Taiwan are divided into four strata according to publications of the Taiwan National Health Research Institute; level 1 indicates the most urbanized areas, and level 4 indicates the least urbanized areas.

^§^DM: diabetes mellitus; HIV: human immunodeficiency virus infection; transplant: operation of transplant; ESRD: end stage renal disease; HTN: hypertension; CHC: chronic hepatitis and cirrhosis.

**Table 2:** Sensitivity analysis of the adjusted odds ratios of diabetes in risk of Kaposi's sarcoma

	DM	
	OR	95%CI
Main model^*^	2.26	1.68 to 3.04
Additional covariates^†^		
Main model + ESRD	2.15	1.59 to 2.91
Main model + HTN	1.94	1.42 to 2.64
Main model + CHC	2.08	1.54 to 2.81
Subgroup effects		
Sex		
Male	2.09	1.49 to 2.93
Female	3.15	1.63 to 6.08
Age		
<70	2.12	1.19 to 3.78
≥70	2.29	1.62 to 3.25
Medical diseases		
ESRD		
Yes	>999	<.01 to >999
No	2.15	1.59 to 2.91
HTN		
Yes	1.98	1.39 to 2.81
No	1.81	0.93 to 3.54
CHC		
Yes	1.28	0.78 to 2.08
No	2.83	1.93 to 4.14

*The main model was adjusted for age, sex, income, urbanization, HIV and transplant.

^†^Models were adjusted for covariates in the main model as well as each additional listed covariate.

### Activation of KSHV lytic program by high glucose

**Figure 1:**
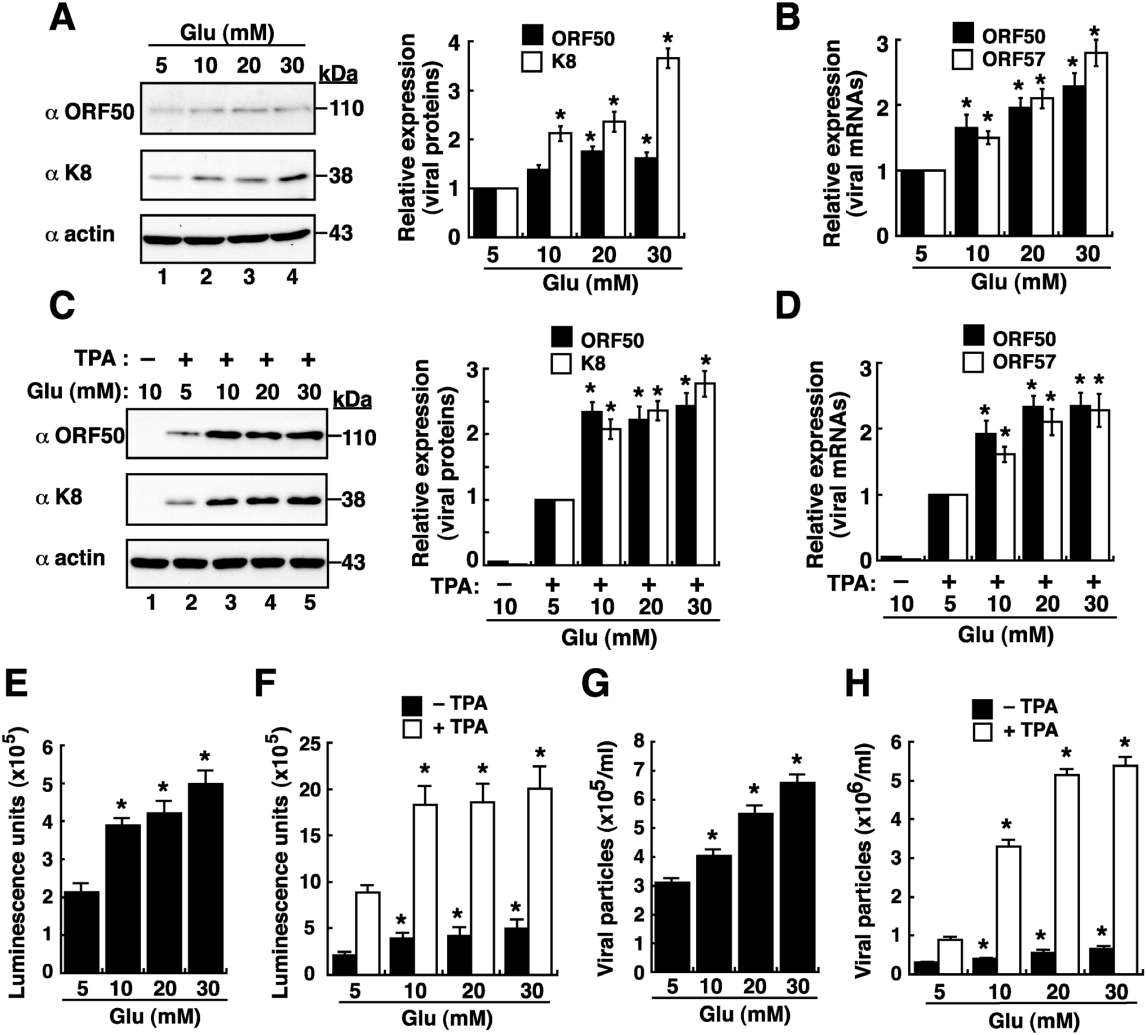
High glucose enhances lytic reactivation of KSHV in BCBL1 cells **(A)** Western blot analysis of viral lytic protein expression in BCBL1 cells that were cultured in various glucose concentrations for 48 hours. Relative expression levels of ORF50 and K8, normalized to β-actin, in BCBL1 cells were shown in the right panel (n=3). **(B)** Quantitative examination of ORF50 and ORF57 mRNAs in BCBL1 cells exposed to different glucose concentrations. Relative levels of ORF50 and ORF57 mRNAs, normalized to the GAPDH mRNA, were determined (n=3). **(C)** Western blot analysis of viral lytic protein expression in BCBL1 cells after treatment with TPA (15 ng/ml) and glucose at various concentrations for 48 hours. Relative expression levels of ORF50 and K8, normalized to β-actin, in BCBL1 cells were shown (right panel, n=3). **(D)** Expression of ORF50 and ORF57 mRNAs in BCBL1 cells after treatment with TPA (15 ng/ml) and glucose at various concentrations for 48 hours (n= 3). **(E and F)** Activation of the ORF50 promoter (ORF50p)-driven luciferase reporter by high glucose in BCBL1 cells in the absence or presence of TPA (n=3). The reporter plasmid pORF50p(−3801/+10) was used in transfection, and the transfected cells were cultured in different glucose concentrations in the absence or presence of TPA for 48 hours. Luciferase reporter assays were performed as described in Materials and Methods. **(G and H)** Virus particles released from BCBL1 cells after exposure to different glucose concentrations without or with TPA for 3 days (n=3). Data are presented as mean ± SEM. Symbol * indicates significant difference vs. the normal glucose treatment (P < 0.05).

As a high level of blood sugar is a hallmark of diabetes, we initially investigated whether high glucose induced KSHV reactivation in latently infected cell line BCBL1. Notably, although KSHV in BCBL1 cells is predominantly latent, a small cell proportion (1∼3%) may undergo spontaneous viral reactivation under the normal culture condition. When BCBL1 cells were cultured in media with elevated concentrations of glucose for 48 hours, high glucose conditions (10, 20 and 30 mM) substantially increased the expression levels of viral lytic proteins such as ORF50 and K8 by 1.5–3.5 fold as compared to the normal glucose condition (5 mM) (Figure 1A). Consistent with the Western blot analysis, quantitative RT-PCR revealed that high glucose increased levels of ORF50 and ORF57 mRNAs, two viral lytic gene transcripts, in a dose-dependent manner (Figure 1B). In combination with a suboptimal dose of a lytic inducing agent TPA, high glucose also enhanced the expression of viral lytic proteins and mRNAs in BCBL1 cells as detected by Western blotting (Figure 1C) and by quantitative RT-PCR (Figure 1D), respectively.

Since viral protein ORF50 is the key latent-to-lytic switch activator in the lytic activation of KSHV [21, 22], its transcriptional activation was examined in response to high glucose. A luciferase reporter plasmid pORF50p(−3801/+10)/luc that contains a 3.8-kb ORF50 gene promoter (ORF50p) was used in transient transfection assays. Compared to the transfected cells cultured in normal glucose, high glucose cultures (10, 20 and 30 mM) significantly increased the ORF50p activity in a dose-dependent fashion (Figure 1E). Similarly, high glucose also augmented the promoting effect of TPA on the ORF50p activity in BCBL1 cells (Figure 1F). To further understand whether high glucose induced the entire viral lytic cycle to completion, virus particles released from BCBL1 cells were measured. Quantitative analysis displayed that the amounts of virus particles released from BCBL1 cells were also significantly increased with elevated concentrations of glucose, regardless of the presence or absence of TPA treatment (Figure 1G and 1H).

**Figure 2:**
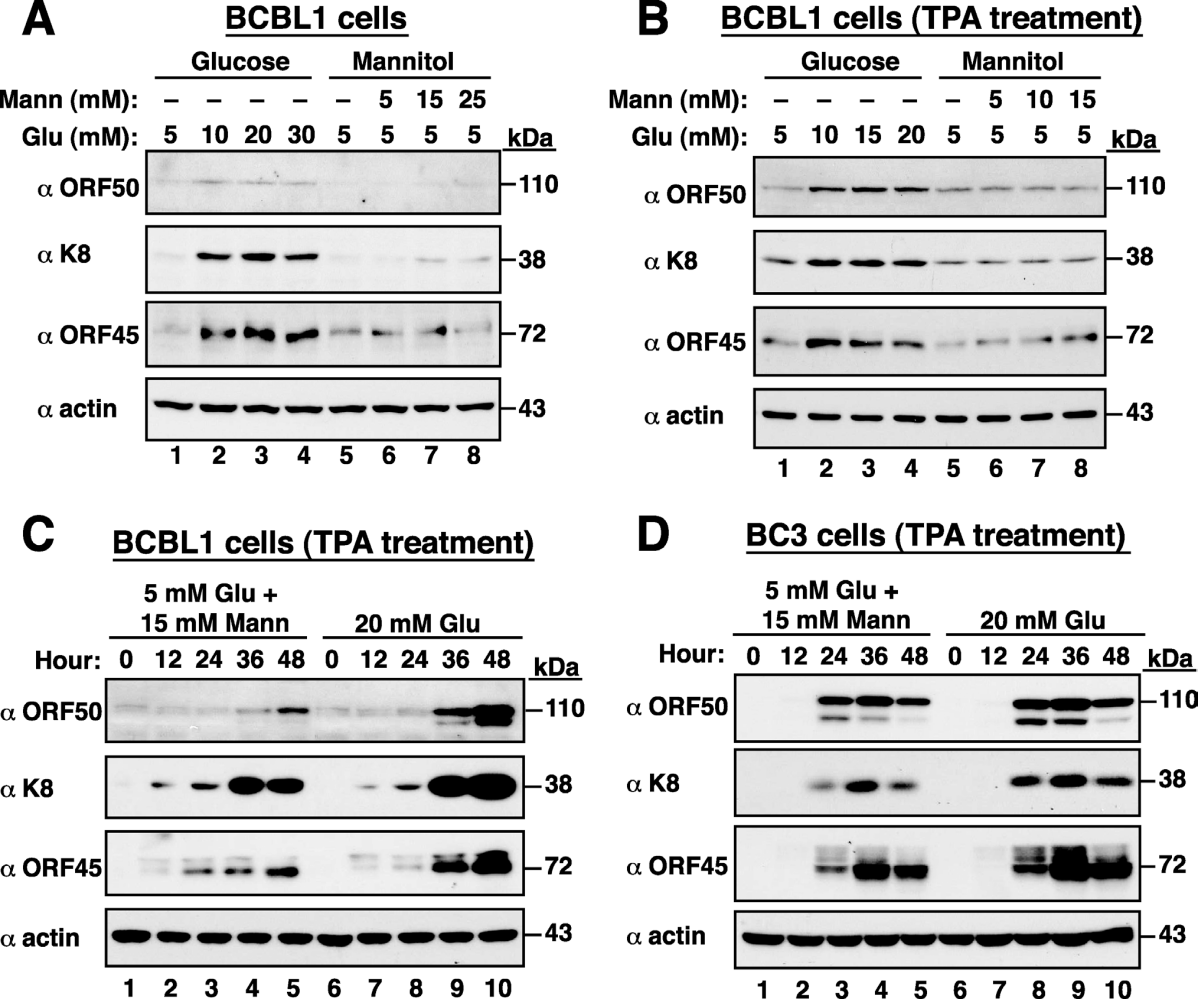
High glucose but not mannitol substantially increases KSHV reactivation **(A)** Expression of viral lytic proteins in BCBL1 cells that were cultured in high glucose or mannitol for 48 hours. **(B)** BCBL1 cells were treated with a combination of TPA (15 ng/ml) and high glucose or mannitol for 36 hours. Viral lytic proteins were analyzed by Western blot analysis. **(C)** Time course analysis of viral lytic protein expression in TPA-induced BCBL1 cells that were cultured in high glucose or mannitol. **(D)** Time course analysis of viral lytic protein expression in TPA-induced BC3 cells that were exposed to high glucose or mannitol.

To rule out the possible osmotic effects of high glucose on KSHV reactivation, BCBL1 cells treated with mannitol served as the osmotic control. We found that high glucose but not mannitol substantially increased the expression levels of viral lytic proteins including ORF50, K8 and ORF45 in BCBL1 cells either untreated or treated with TPA (Figure 2A and 2B). Time course experiments also demonstrated that high glucose but not mannitol promoted TPA-mediated viral reactivation in BCBL1 cells (Figure 2C). In addition to BCBL1 cells, a similar promoting effect of high glucose on the progression of viral reactivation was also observed in another KSHV-positive cell line BC3 (Figure 2D).

**Figure 3:**
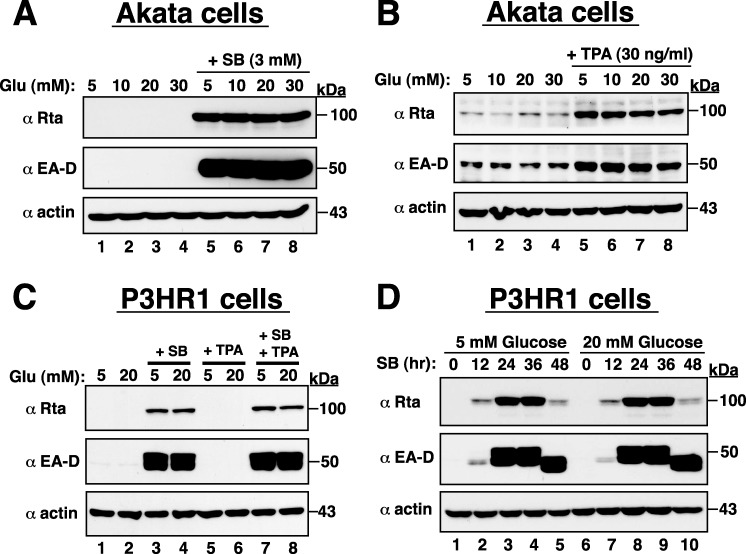
High glucose cannot promote EBV lytic reactivation in infected cells **(A and B)** Akata cells exposed to different glucose concentrations alone or in combination with SB or TPA were prepared and subjected to Western blot analysis. Rta and EA-D are two early-lytic proteins of EBV. **(C)** Effect of high glucose on lytic protein expression of EBV in P3HR1 cells under the latent condition or under the pro-lytic conditions with SB, TPA or both SB and TPA treatment. **(D)** Time course analysis of EBV lytic protein expression in SB-treated P3HR1 cells cultured in normal or high glucose.

Due to many broad similarities between KSHV and Epstein-Barr virus (EBV), we also investigated whether high glucose reactivated EBV from latency. Two naturally EBV-infected cell lines, Akata and P3HR1, were used in the study. Results from Western blot analysis showed that high glucose did not affect the expression of EBV lytic proteins such as Rta and EA-D in both Akata and P3HR1 cells, either under the latent condition or under the lytic culture conditions (treatment with TPA or sodium butyrate) (Figure 3). These results indicate that high glucose specifically induces lytic reactivation of KSHV but not EBV, and emphasize that KSHV and EBV may use different regulatory pathways to control their latent-to-lytic switch in infected cells.

### Involvement of AP1 in high glucose-mediated viral reactivation

**Figure 4:**
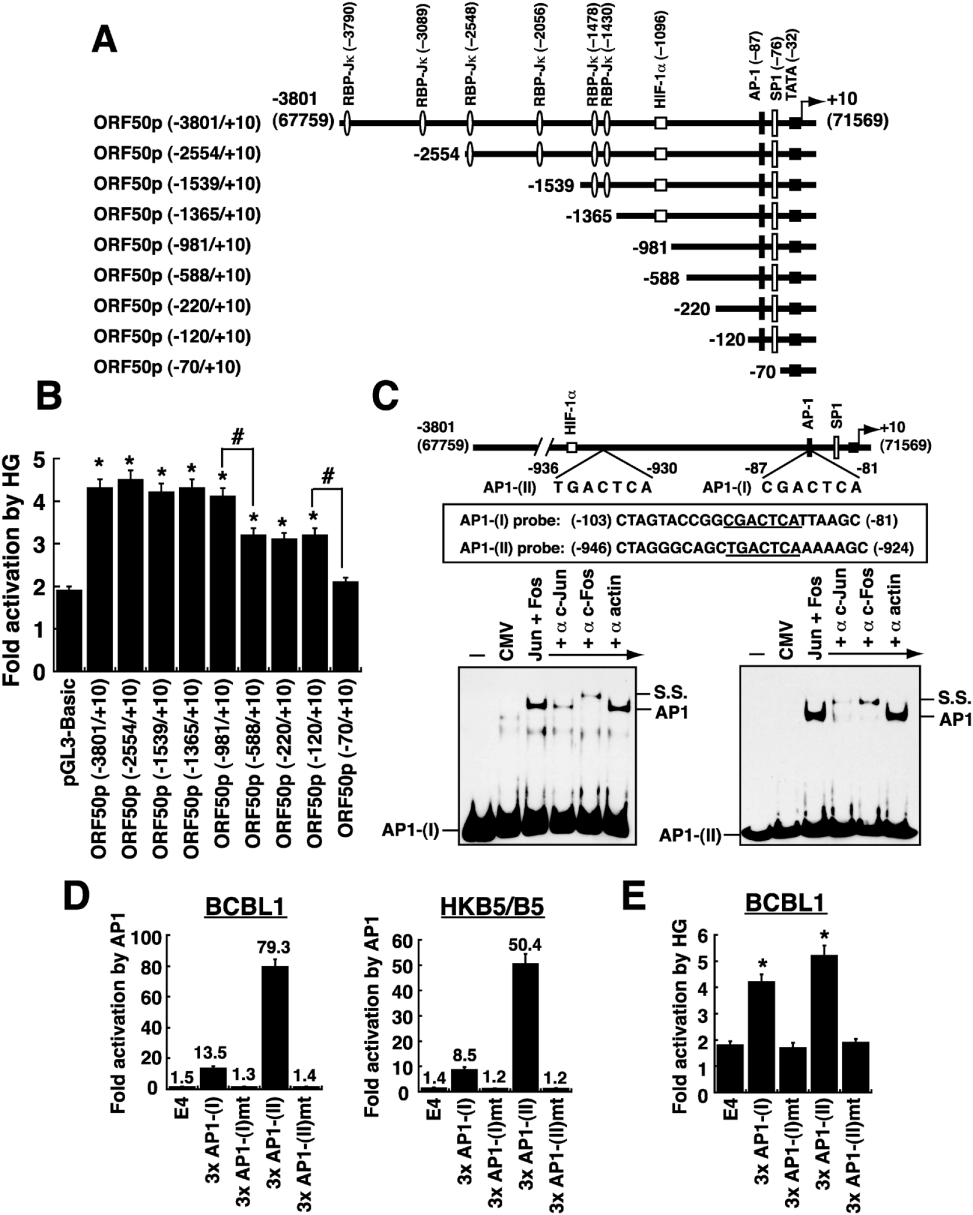
Defining the critical response elements in the ORF50 promoter to high glucose **(A)** Schematic diagram of ORF50p deletion reporter constructs. Several known binding sites for transcription factors such as RBP-Jκ, HIF-1α, AP-1 and SP1 in the ORF50 promoter are shown in the diagram. **(B)** Responsiveness of the ORF50p deletions to high glucose. BCBL1 cells were transfected with the indicated reporter plasmids, and then the transfected cells were cultured in normal or high glucose (20 mM). The fold activation of each reporter plasmid that responds to high glucose was calculated as described in Materials and Methods. Data are indicated as mean ± SEM (n=4). Symbol * represents significant difference vs. pGL3-Basic, and symbol # represents significant difference vs. the indicated deletion constructs (P < 0.05). **(C)** Direct binding of AP1 protein complex (c-Jun and c-Fos) to AP1-(I) element and AP1-(II) element. EMSA experiments were performed using protein extracts of 293T cells transfected with empty vector or transfected with plasmids expressing c-Jun and c-Fos. Antibodies to c-Jun and c-Fos were used to supershift or remove the formed complex. The AP1-specific complex and the supershifted (SS) complex are indicated. **(D)** Activation of AP1-(I)- and AP1-(II)-containing reporter constructs by AP1 protein complex (n=3). The reporter plasmids containing 3 copies of the wild-type or mutated AP1-(I) element or AP1-(II) element were individually cotransfected with plasmids expressing c-Jun and c-Fos into BCBL1 cells or HKB5/B5 cells. Luciferase activity in these transfected cells was measured 24 hours after transfection. **(E)** Responsiveness of the AP1-(I) and AP1-(II) elements to high glucose in BCBL1 cells. Transient reporter assay was performed in BCBL1 cells that were transfected with the indicated reporter plasmids. After the transfected cells were cultured in either normal glucose or high glucose (20 mM) for 48 hours, the response of each reporter construct to high glucose was determined. Data are represented as mean ± SEM (n=4). Symbol * represents significant difference vs. pE4luc (P < 0.05).

Since the switch between latency and lytic replication of KSHV is initiated by the expression of ORF50 protein [21, 22], we further characterized its transcriptional regulation in response to high glucose. A series of deleted ORF50p regions linked to luciferase gene were constructed (Figure 4A) and the resultant reporter plasmids were individually transfected into BCBL1 cells. After the transfected cells were cultured in normal or high glucose (20 mM) for 2 days, we consistently found that two promoter regions from −981 to −588 and from −120 to −70 were important for the response to high glucose (Figure 4B). In the promoter region from −120 to −70, it contains a known AP1-binding site and an SP1-binding site, which reportedly confer the response to TPA and sodium butyrate (SB), respectively [23, 24]. However, the importance of the promoter region from −981 to −588 for the ORF50p activation has never been mentioned before. Sequence analysis showed that a perfect consensus AP1-binding site, TGACTCA, is located between −930 and −936 (Figure 4C). Here, we designated the previously identified AP1 site (–81 to −87) as the “AP1-(I)” element, and the newly identified AP1 site (–930 to −936) as the “AP1-(II)” element.

To determine whether the newly identified AP1-(II) site was functional, we did EMSA experiments. Like the AP1-(I) element, the AP1-(II) element was bound by AP1 protein complex (c-Jun and c-Fos) in EMSAs (Figure 4C). Furthermore, point mutations at the AP1-(II) site in the ORF50 promoter substantially impaired the promoter activation by AP1 in BCBL1 and HKB5/B5 cells (Supplementary Figure 2). Particularly, we showed that overexpression of AP1 activated the reporter plasmid that encompasses three copies of the wild-type AP1-(II) element [p3xAP1-(II)/E4luc], but not the AP1-(II)-mutated reporter plasmid [p3xAP1-(II)mt/E4luc], in BCBL1 and HKB5/B5 cells (Figure 4D). All these results confirmed that the AP1-(II) element is a functional AP1 target site.

To analyze whether the AP1-(I) or AP1-(II) element conferred the response to high glucose, the reporter plasmids encompassing 3 copies of wild-type or mutated AP1-(I) element or AP1-(II) element were transfected into BCBL1 cells. We found that high glucose activated only the reporter plasmids containing wild-type AP1-(I) or AP1-(II) element but not the mutated reporter plasmids (Figure 4E). In addition to the AP1-containing reporter plasmids, the reporter plasmids with tandem copies of RBP-Jκ- or Sp1-binding element from the ORF50 promoter were also tested for their ability to respond to high glucose in BCBL1 cells (Supplementary Figure 3). Unlike the AP1-containing reporter constructs, high glucose could not activate the reporter constructs encompassing RBP-Jκ- or Sp1-binding element (Supplementary Figure 3). Our results therefore demonstrated that the two AP1-binding sites from −81 to −87 and from −930 to −936 within the ORF50 promoter are critically required for the response to high glucose.

### Correlation between c-Jun levels and KSHV reactivation under high glucose conditions

**Figure 5:**
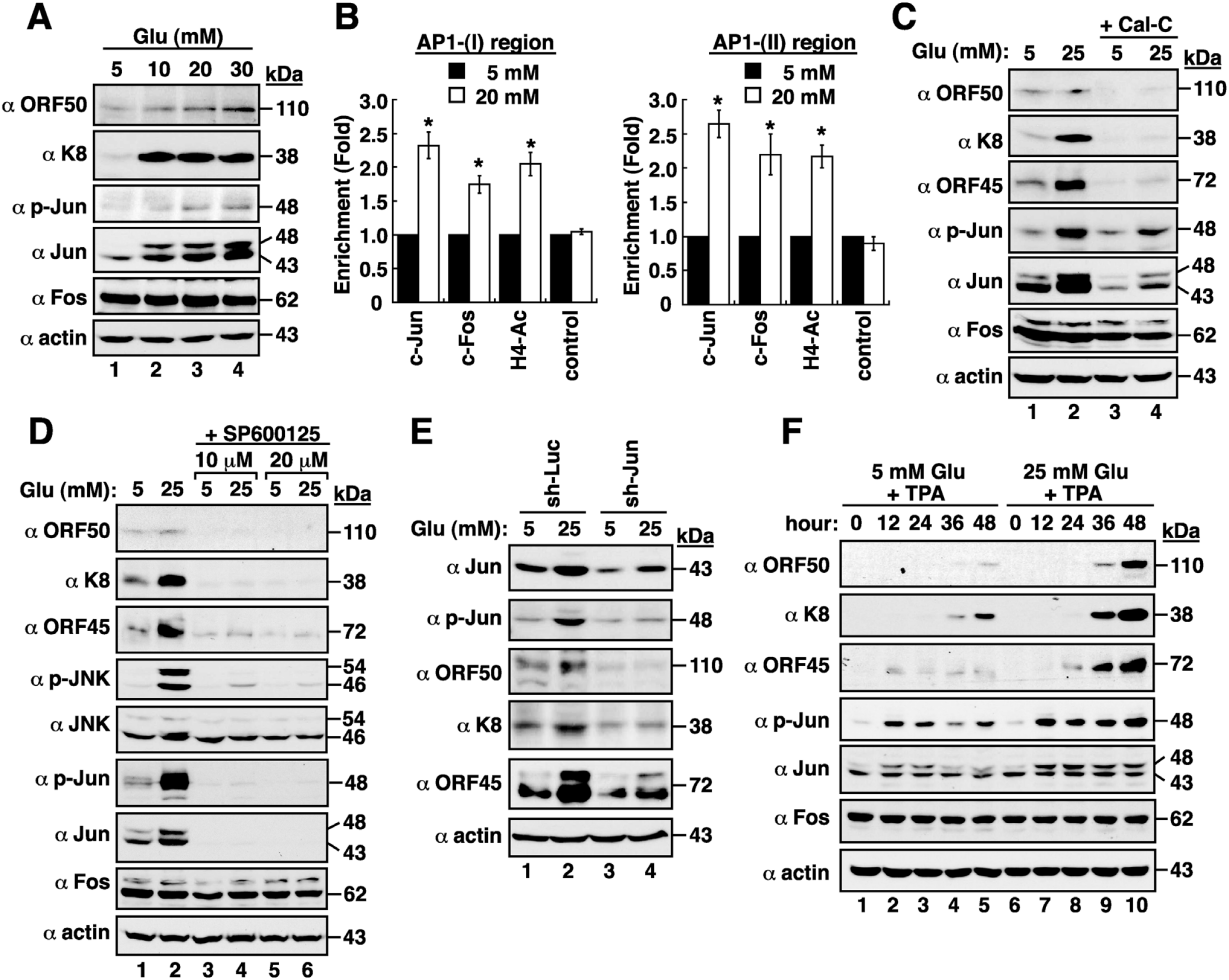
High glucose activates AP1 transcription factor in BCBL1 cells **(A)** Expressions of viral and cellular proteins in BCBL1 cells that were cultured at different glucose concentrations. **(B)** Chromatin immunoprecipitation (ChIP) assays were performed to evaluate the binding of c-Jun, c-Fos, or acetyl-histone H4 to the AP1-(I)- or AP1-(II)-containing region of the ORF50 promoter in normal and high-glucose cultured BCBL1 cells. Data are represented as mean ± SEM (n=3). Symbol * represents significant difference vs. the normal glucose treatment (P < 0.05). **(C)** Effect of calphostin C, a PKC inhibitor, on viral reactivation. BCBL1 cells that were untreated or treated with calphostin C (Cal-C; 0.25 μM) were cultured in media containing normal or high glucose (25 mM) for 2 days. The treated samples were subjected to Western blot analysis. **(D)** Effect of SP600125, a JNK inhibitor, on high glucose-mediated viral reactivation. SP600125 at a concentration of 10 μM or 20 μM was used to treat BCBL1 cells for 2 days under normal or high glucose (25 mM) conditions. **(E)** Effect of c-Jun knockdown on high glucose-mediated viral reactivation. BCBL1 cells that were infected with lentivirus encoding shRNA to luciferase (Luc) or to c-Jun were cultured in normal or high glucose conditions. At 24 hr after lentiviral infection, cells were harvested and analyzed by immunoblotting using the indicated antibodies. **(F)** Time course analysis of AP1 components and viral lytic proteins expressed in TPA-induced BCBL1 cells that were exposed to normal or high glucose.

We next examined whether the expression levels of individual AP1 components, such as c-Jun and c-Fos, were affected by high glucose in BCBL1 cells. Western blot analysis revealed that high glucose did not alter c-Fos expression in cells; however, both total c-Jun and phosphorylated c-Jun levels were evidently increased by high glucose (Figure 5A). We then evaluated whether the binding of AP1 to ORF50 promoter in BCBL1 cells was enhanced by high glucose. Results from chromatin immunoprecipitation (ChIP) assays revealed that high glucose treatment indeed increased the binding of the AP1 protein complex to the AP1-(I)- and AP1-(II)-containing regions in the ORF50 promoter (Figure 5B). Furthermore, treatment of BCBL1 cells with calphostin C (Cal-C; a PKC inhibitor), SP600125 (a JNK inhibitor) or a lentivirus-based vector expressing c-Jun shRNA, which decreased both total c-Jun and phosphorylated c-Jun levels, also led to reduced viral lytic protein expression (Figure 5C–5E). In combining with TPA, high glucose also promoted the levels of total c-Jun and phosphorylated c-Jun in BCBL1 cells during viral lytic induction (Figure 5F), which correlated with increased levels of viral lytic protein expression. These results suggested that active AP1 protein complex caused by high glucose significantly contributes to the enhancement of KSHV reactivation.

### Effect of high glucose on target cell susceptibility to KSHV infection

**Figure 6:**
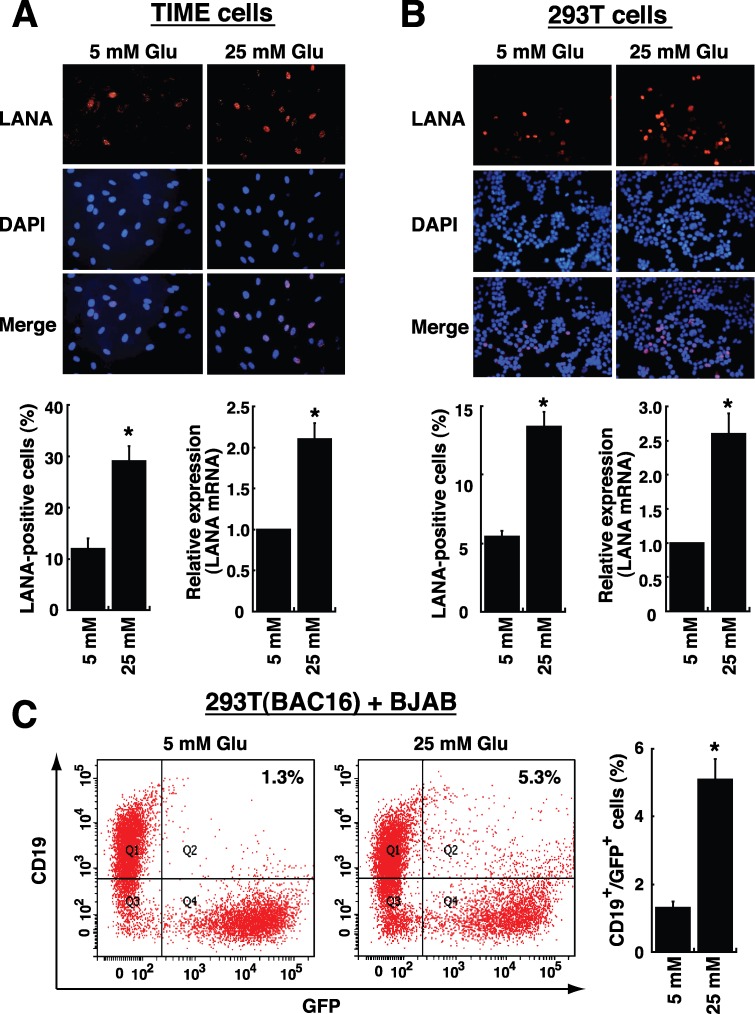
High glucose increases cell susceptibility to KSHV infection TIME cells **(A)** or 293T cells **(B)** were cultured under normal (5 mM) or high glucose (25 mM) conditions for 2 days, and then infected with KSHV for 2 hours. After removing unbound viruses, cells were cultured for another 24 hours in normal or high glucose. Representative images of LANA immunofluorescence staining are shown in the upper panel. The calculated percentages of LANA-positive cells (n= 5) and the relative mRNA levels of LANA (n= 3) in infected TIME cells and 293T cells are showed in bottom panels. **(C)** BJAB cells were cocultured with 293T(BAC16) cell in a 3:1 ratio, and the cocultures were exposed to normal glucose or high glucose for 4 days. The percentages of BJAB cells infected with BAC16-KSHV (CD19 and GFP double-positive cells) were examined by flow cytometry (n= 3). All data are represented as mean ± SEM. Symbol * represents significant difference vs. the normal glucose treatment (P < 0.05).

To investigate whether high glucose modulated cell susceptibility to viral infection, TIME (endothelial) cells and 293T (epithelial) cells were used as target host cells. TIME and 293T cells were first pre-cultured in media with normal or high glucose (25 mM) for 2 days, and then the pre-treated cells were incubated with KSHV virions for 2 hours. After the unbound virions were washed out, the cultures were continued for 1 day with media containing normal or high glucose. To detect viral infection, the expression of viral latency-associated nuclear antigen (LANA) [2] in infected cells was examined by immunofluorescence and by quantitative RT-PCR. Immunofluorescence analysis showed that TIME or 293T cells cultured in high glucose had a nearly 2.5-fold increase in viral infection compared to these cells cultured in normal glucose (Figure 6A and 6B). Similar results were also obtained using RT-PCR to quantify LANA mRNA levels in TIME and 293T cells (Figure 6A and 6B). In addition to TIME and 293T cells, a B lymphoma cell line BJAB was also used for KSHV infection. Since B cells were refractory to infection with soluble KSHV virions [25], a cell-to-cell coculture system was utilized to analyze KSHV transmission from an infected cells to B cells. In the case, BJAB cells were mixed with 293T(BAC16) cells, a 293T cell clone containing the BAC16-derived KSHV genome and a green fluorescent protein (GFP) gene in the viral genome. After cells were cocultured for 4 days in normal or high glucose-containing media, we found that the cocultures in high glucose caused almost a 4-fold increase in the proportions of CD19^+^/GFP^+^ cells compared to the cocultures in normal glucose (Figure 6C).

### Effect of high glucose on the expression of cellular receptors for KSHV infection

**Figure 7:**
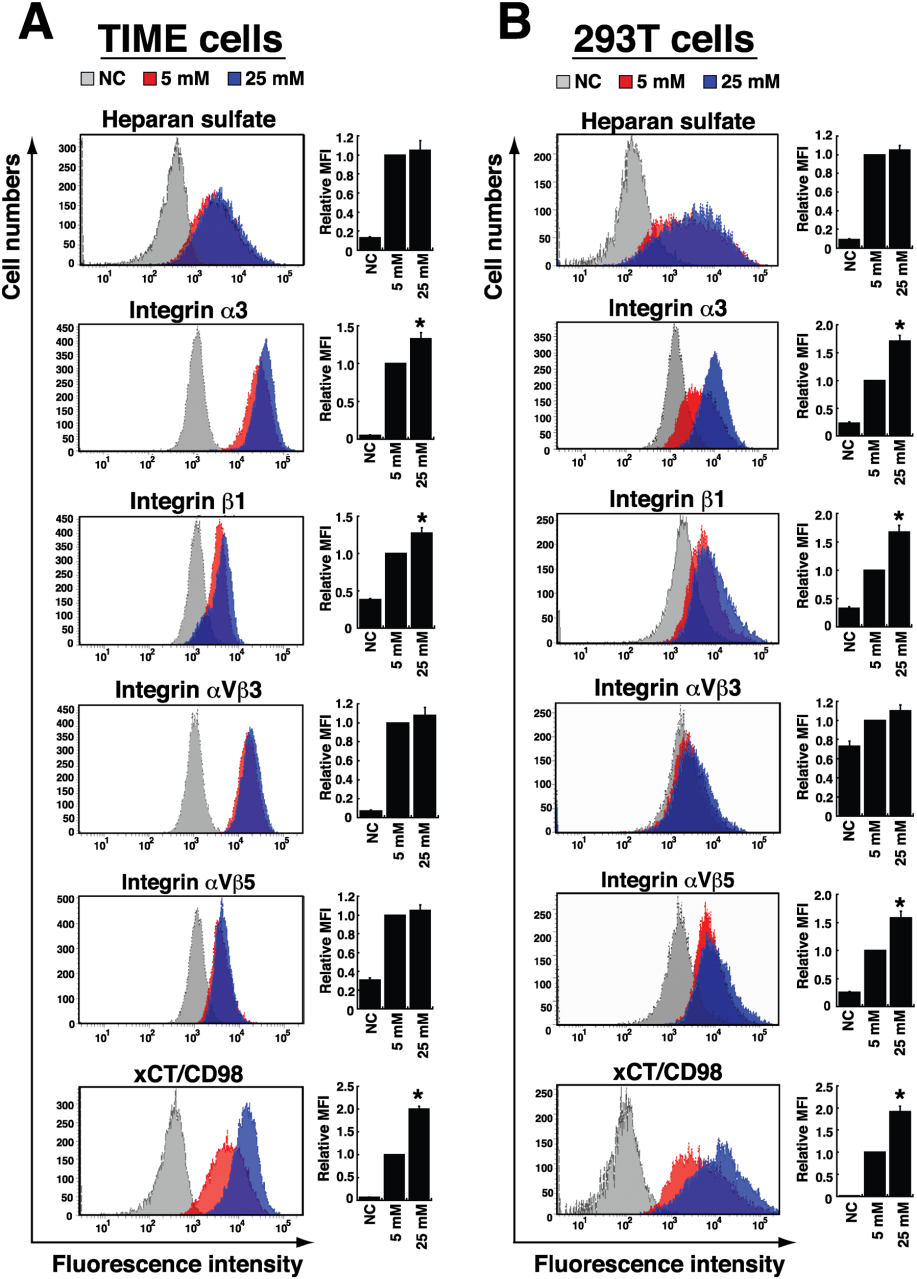
Effects of high glucose on the expression of cellular receptors for KSHV binding and entry TIME cells **(A)** and 293T cells **(B)** were cultured in normal (5 mM) or high glucose (25 mM) for 4 days and 2 days, respectively. Cells were stained with specific antibodies to heparan sulfate, integrins α3, β1, αVβ3, and αVβ5, as well as xCT/CD98, and analyzed by flow cytometry. Bar graphs represent relative MFI (mean fluorescent intensity) of individual KSHV-binding or entry receptors expressed in cells cultured in normal or high glucose (n=3). NC: isotype negative control. Symbol * indicates significant difference vs. the normal glucose treatment (P < 0.05).

As we know that the interaction between viral glycoproteins and cellular receptors on target cell surface is a critical step for KSHV infection, we determined whether high glucose affected the expression of cellular receptors for KSHV binding and entry. In TIME and 293T cells, heparan sulfate, integrins α3β1, αVβ3, αVβ5 and xCT/CD98 are known as the major viral binding or entry receptors [26, 27]. We therefore assessed their expression in response to high glucose. Flow cytometric analysis revealed that high glucose treatment (25 mM) did not affect expression levels of heparan sulfate and integrin αVβ3 in either TIME or 293T cells (Figure 7). However, in TIME cells, we found that high glucose appeared to increase levels of integrin α3, integrin β1 and xCT/CD98 by 1.3-, 1.3- and 2.0-fold, respectively (Figure 7A). Similarly, 293T cells cultured in high glucose also had higher expression levels of integrin α3, integrin β1 and xCT/CD98 (1.7-, 1.7- and 1.9-fold, respectively) than 293T cells cultured in the normal glucose condition (Figure 7B). Unlike TIME cells, 293T cells cultured in high glucose showed an increased level of integrin αVβ5 (by 1.6-fold). Collectively, these results suggest that elevated levels of specific KSHV entry receptors caused by high glucose may be linked to increased susceptibility of TIME and 293T cells to KSHV infection.

## DISCUSSION

Although people with diabetes are common at high risk for the growth of certain tumors, the causal relationship between diabetes and KS actually remains obscure. Until now, only two case-control studies proposed that diabetes could be a risk factor for KS. In the first study, Guttman-Yassky et al. [28] enrolled 35 KS cases and 48 matched KSHV-infected controls in Israel. Although the increased odds of KS among patients with diabetes did not reach statistical significance (OR = 1.78; 95% CI, 0.43 to 7.36), they still proposed that diabetes could promote KS. In the second study, Anderson et al. [9] recruited 142 KS cases and 123 KSHV-infected healthy controls in Sicily, and showed that diabetes was associated with an OR of 4.73 (95% CI, 2.02 to 11.1) for KS occurrence. Due to considerable geographical variation in KSHV prevalence, the association between diabetes and KS in different populations needs to be further evaluated. In Taiwan, KSHV seroprevalence was estimated to be 13.4% to 22.8% in adults [29–31]. According to our dataset including 352 KS patients and 1, 408 matched controls, we here show a meaningful association of diabetes with an increased risk of KS (OR = 2.25; 95% CI, 1.68 to 3.04) (Tables 1 and 2). Noteworthily, most of diabetic cases enrolled in the study are found to be type 2 DM (192 out of 193 cases in the non-KS group and 86 out of 87 cases in the KS group). Furthermore, based on data from the medical records of these diabetes-associated KS patients (87 cases), the mean duration of the time interval from the clinical diagnosis of diabetes to KS occurrence is 4.1 years with a stand deviation of 3.22 years (Supplementary Figure 4). Since certain anti-diabetes medications might potentially promote or delay the occurrence of KS, we also analyzed the relationship between types of anti-diabetes medications (including insulin, metformin, sulfonylureas, meglitinides, thiazolidinediones, α-glucosidase inhibitors, and dipeptidyl petidase-4 inhibitors) and KS occurrence. As shown in Supplementary Table 1 and Supplementary Figure 5, we found that there is no statistically significant difference between types of anti-diabetes therapy and KS occurrence.

Since all subtypes of KS are linked to KSHV infection [2] and active viral lytic program strongly correlates with KS progression and severity [2–5], certain characteristics in diabetes may be critical for KS development by promoting viral lytic reactivation and viral infection. Three reasons prompt us to continuously ask whether high glucose can be a physiological stimulus for KSHV reactivation or KSHV infection. Firstly, the presence of high glucose concentration in blood is the hallmark of diabetes, and our case-control studies have already provided evidence showing the association of diabetes with increased risk of KS (Table 1). Secondly, many cell signalling transducers or effectors activated by high glucose often overlap with those involved in the regulation of KSHV reactivation. Thirdly, glucose can be considered as a “hormone”, which may modulate cellular metabolism to facilitate both latent and lytic KSHV infection. Due to the fact that diabetes is a chronic disease and is often diagnosed several years after onset, we here used glucose at high doses (10, 20 and 30 mM) in our *in vitro* studies to mimic the diabetic/hyperglycemia conditions [12–16]. The concentrations of 10, 20 and 30 mM glucose equivalent to 180, 360 and 540 mg/dl, respectively, are actually higher than the physio-pathological concentrations in diabetics (Fasting plasma glucose: >126 mg/dl; Random plasma glucose: >200 mg/dl). However, based on the previously reported literatures [12–16], most laboratories used 5 mM glucose concentration (90 mg/dl) as a control condition and 25 mM glucose concentration (450 mg/dl) as a hyperglycemic attack for their *in vitro* studies. Our present studies also routinely include these two widely used concentrations of glucose (5 mM and 25 mM) for our further experiments. In the study, we indeed demonstrated that high glucose but not mannitol increases KSHV reactivation in latently infected BCBL1 cells (Figures 1 and 2). The positive effect of high glucose on KSHV reactivation in BCBL1 cells was not due to the altered cell proliferation because we did not detect significant changes in cell numbers of BCBL1 cells that were cultured in different glucose concentrations (5, 10, 20 and 30 mM) during a 3-day culture period (Supplementary Figure 6A). Furthermore, in the presence of other lytic inducing stimuli (e.g. TPA), we showed that high glucose still augmented the promoting effect of the inducing stimulus on viral reactivation (Figures 1 and 2). In contrast, although EBV is also a human oncogenic herpesvirus most closely related to KSHV, we did not detect the similar lytic activation by high glucose in infected cells under latent or lytic culture conditions (Figure 3). These findings implicate that high glucose may specifically affect KSHV-associated malignancies but not EBV-associated malignancies.

As the switch between latency and lytic-gene expression of KSHV is initiated by the expression of the ORF50 gene, we focus particularly on the transcriptional regulation of the ORF50 gene promoter by high glucose. Based on reporter assays and ChIP analyses, we showed that active AP1 (Jun/Fos) protein complex binding to the ORF50 promoter specifically triggers the expression of ORF50 protein under high glucose conditions (Figures 4 and 5), thereby leading to increased viral lytic gene expression. Importantly, we uncover another functional AP1-binding site in the ORF50 promoter, which is located between −936 and −930 (Figure 4). On the other hand, although Ye et al. [20] reported that high glucose could activate ERK, p38 and JNK pathways in KSHV-infected cells, we here demonstrated that activation of PKC, JNK and c-Jun by high glucose is critically involved in KSHV lytic reactivation (Figure 5C–5E).

In addition to viral reactivation, periodic re-infection of KSHV to new target cells appears to be relevant to increase the risk of KS development. KSHV can infect a variety of target cells including endothelial cells, epithelial cells, and B cells [27], which may alter the metabolic profiling and oncogenic behavior of these infected cells and consequently lead to an increase in glucose uptake and consumption [32, 33]. We here showed that high glucose significantly increases the susceptibility of TIME and 293T cells to KSHV infection (Figure 6A and 6B). Furthermore, although the major routes of KSHV infection to B cells could be quite different from the routes to endothelial and epithelial cells [25, 26], our results showed that high glucose enhances B cells infected with KSHV in a cell-to-cell cocultivation system (Figure 6C). These findings suggest that high glucose microenvironment is capable of modulating cellular functions in different target cells, thereby increasing their susceptibility to KSHV infection. During KSHV infection, the interactions between viral glycoproteins and cellular receptors are important not only for viral attachment and entry, but also for the outside-in signal transduction and the subsequent modulation of viral gene expression [26, 27]. Although the cell growth rates of both TIME and 293T cells were found to be different under high glucose conditions (Supplementary Figure 6B and 6C), we consistently found that the expressions of specific KSHV entry receptors including integrin α3, β1, and xCT/CD98 were significantly increased in both TIME and 293T cells under high glucose conditions (Figure 7). Interestingly, studies on diabetic retinopathy, Part et al. also reported that high glucose could increase the expression of integrin α3 and β1 in retinal capillary pericytes [34]. Additionally, several lines of evidence revealed that the temporal interaction between integrins α3β1 and xCT/CD98 is relevant to cell adhesion, fusion, proliferation and the integrin-dependent signal transduction [26, 35]. Noteworthily, the xCT/CD98 complex, a cystine/glutamate exchange transporter, naturally functions to maintain the intracellular redox balance and protects cells from death induced by oxidative stress [35, 36]. Based on the mentioned roles of integrins α3β1 and xCT/CD98 in cells, we therefore propose that elevated levels of integrin α3β1 and xCT/CD98 in endothelial and epithelial cells caused by high glucose may facilitate subsequent KSHV infection. To our knowledge, this is the first work showing that environmental stimuli (i.e., high glucose) can activate a particular set of KSHV entry receptors in target cells.

In conclusion, our studies propose a positive correlation between diabetes and the risk of KS. Identification of the potential stimulating cofactors for KS may allow us to provide an effective intervention strategy early on the development of KS.

## MATERIALS AND METHODS

### Databases and study design

Two subparts of the Taiwan National Health Insurance Research Database (NHIRD) were used in a case-control study, including Registry of Catastrophic Illness Patient Database (RCIPD) and Longitudinal Health Insurance Database 2005 (LHID 2005). The RCIPD contains all confirmed cases of catastrophic illness, including KS and other cancers. The LHID 2005 was compiled from a random sampling of 1 million individuals, who were enrolled in 2005, from the total population contained in the NHIRD. Patients with KS (ICD-9 code176) were identified from the RCIPD between 1997 and 2008 as a case group and a matched control group was identified from the LHID 2005. For each KS case, 4 cancer-free controls that were individually matched to the case on gender, age, monthly income, and urbanization levels were enrolled. The sampling procedures are summarized in Supplementary Figure 1. A total of 1, 760 patients (352 KS cases and 1, 408 controls) were included in final analysis. Potential confounding risk factors for KS were identified as the following diagnoses: diabetes (ICD-9 code 250), HIV infection (HIV; ICD-9 codes 042–044 and V08), and operation of transplant (ICD-9 codes V420, V421, V426, V427 and V4283). Additionally, end stage renal disease (ESRD; ICD-9 code 585.6), hypertension (HTN; ICD-9 codes 401–405), and chronic hepatitis and cirrhosis (CHC; ICD-9 code 571) were also included as covariates in the sensitivity analysis. The study was exempted from approval by the Institutional Review Board of Chang Gung Memorial Hospital (IRB# 20161044B1).

### Cell cultures and transfections

BCBL1 [37] and BC3 [38], two KSHV-positive lymphoma cell lines, were cultured in RPMI 1640 medium with 15% fetal bovine serum (FBS). Akata and P3HR1 cells, two EBV-positive lymphoma cell lines, were obtained from Dr. Shih-Tung Liu (Chang-Gung University, Taiwan). These two EBV-positive cells were grown in RPMI1640 medium with 10% FBS. TIME cells [39], a dermal microvascular endothelial cell line, were cultured in Medium 199 supplemented with 20% FBS and 15 ng/ml Endothelial Cell Growth Supplement (ECGS; Merck Millipore). 293T cells were cultured in DME medium with 10% FBS. 293T(BAC16) is a 293T cell clone containing BAC16-KSHV genome. BJAB and HKB5/B5 cells [17] were grown in RPMI 1640 medium with 10% FBS. In some experiments, cells were cultured in high glucose concentrations, and mannitol served as an osmotic control for high glucose. For chemical treatment, 12-*O*-tetradecanoyl-phorbol-13-acetate (TPA)(15 ng/ml or 30 ng/ml), sodium butyrate (3 mM), calphostin C (0.25 μM), and SP600125 (10 μM or 20 μM) were used. Transient transfection experiments were performed with Lipofectamine 2000 (Invitrogen).

### Western blot analysis

Western blot analysis was performed as described previously [17]. The anti-ORF50 antibody used in the study was generated in our laboratory [17]. Antibodies to K8 (sc-57889; Santa Cruz), ORF45 (sc-53883; Santa Cruz), Rta (8C12; Argene), EA-D (sc-58121; Santa Cruz), c-Fos (sc-52; Santa Cruz), c-Jun (#9165; Cell signaling), phospho-c-Jun (#3270; Cell signaling), JNK (sc-474; Santa Cruz), phospho-JNK (#4668; Cell signaling), and actin (MAB101; Chemicon) were obtained commercially.

### Quantitative reverse transcription (RT)-PCR

Total RNA extraction, reverse transcription, and quantitative PCR based on SYBR-Green I fluorescence were performed as mentioned previously [17]. The primer sets were following: 5′-AGATGACAAGGGTAAGAAGC and 5′-CGCACCAAGCTTGGAACATTC for ORF50; 5′-GGACATTATGAAGGGCATCCTAGAG and 5′-GACTGGGTACCACGGATGCGCTCGT for ORF57; 5′-GAAGTGGATTACCCTGTTGTTAGC and 5′-CCTCATACGAACTCCAGGTCTGTG for LANA; 5′-TCATGGGTGTGAACCATGAG and 5′-AGTGATGGCATGGACTGTGG for glyceraldehyde-3-phophate dehydrogenase (GAPDH).

### Plasmid construction

The reporter plasmids pORF50p(−3801/+10)/luc and pE4luc have been previously described [17]. To generate ORF50 promoter deletion constructs, DNA fragments of the indicated size were amplified by PCR and cloned into pGL3-Basic (Promega). To construct the reporter plasmids that contain wild-type or mutated copies of AP1-binding sites, synthetic double-stranded oligonucleotides were cloned upstream of the adenovirus E4 promoter in pE4luc. The plasmids that express c-Jun and c-Fos were purchased from OriGene (Rockville, MD).

### Luciferase reporter assay

BCBL1 or HKB5/B5 cells were transfected with a fixed amount of plasmid DNA. The reporter assays were carried out according to manufacturer's protocol for the luciferase reporter assay system (Promega). The fold activation of each reporter construct was calculated as the luciferase activity in the presence of stimuli (including high glucose) divided by that in the absence of stimuli.

### Quantitative TaqMan PCR

Supernatants from BCBL1 cells cultured in normal or high glucose for 3 days were collected. Viral DNA extraction and the subsequent TaqMan PCR analysis were performed as mentioned in our previous studies [40].

### Chromatin immunoprecipitation (ChIP) assay

ChIP assays were performed as previously described [17]. The chromatin complex immunoprecipitated by antibodies to c-Jun (#9165; Cell signaling), c-Fos (sc-52; Santa Cruz), acetyl-histone H4 (04-886; Upstate), or the control IgG were analyzed using real-time PCR. The PCR primers used in the study were as follows: 5′-TAGGACCCAGCTACAGCTTATCCT and 5′-CATTGCCACCCAGCTACTGGTTTC for the AP1-(I) region; 5′-CACGTTGATCCGGCTTACCGACAGT and 5′-CCTGGAAGAGTATGGCGGACTGTC for the AP1-(II) region.

### Electrophoretic mobility shift assay (EMSA)

The DNA probes were end-labeled with biotin-11-UTP using terminal deoxynucleotidyl transferase (PIERCE). Protein extracts of 293T cells transfected with the c-Fos- and c-Jun-expressing plasmids were prepared as described previously [17]. In EMSA binding reactions, 10 μg of protein extract were mixed with 1 ng DNA probe.

### Lentivirus-based knockdown

The lentivirus-based vectors encoding short hairpin RNAs (shRNAs) were all obtained from the National RNAi Core Facility Platform at the Institute of Molecular Biology/Genomic Research Center, Academia Sinica (Taiwan). The target sequence of the c-Jun shRNA is 5′-ATTCGATCTCATTCAGTATTA. Preparation of shRNA lentiviral particles has been described previously [41].

### Viral infection and immunofluorescence

Supernatants from BC3 cells treated with 3 mM sodium butyrate for 3 days were harvested, filtered through a 0.45 μM filter, and centrifuged for 2 hours at 15, 000 rpm in an SW28 rotor. KSHV virions were re-suspended in PBS, aliquoted, and stored at −70°C. Prior to KSHV infection, TIME or 293T cells were pre-cultured for 2 days in media with normal or high glucose (25 mM). KSHV virions were then inoculated with these cells for 2 hours at 37°C. After washing with PBS to remove unbound virions, cells were cultured in normal or high glucose for another 24 hours. Immunofluorecent staining was performed as described previously [41]. For infecting B cells, BJAB cells were co-cultivated with 293T(BAC16) at a 3:1 ratio in normal or high glucose-containing media for 4 days.

### Flow cytometry analysis

After TIME cells and 293T cells were cultured for 4 days and 2 days, respectively, in normal or high glucose-containing media, cells were stained with antibodies specific to integrin α3 (sc-13545; Santa Cruz), β1 (sc-18887; Santa Cruz), αVβ3 (304402; BioLegend), αVβ5 (MAB1961; Millipore), CD98/xCT (315602; BioLegend), and heparan sulfate (370255-1; AMIBIO). An aliquot of cells was stained with isotype-matched control antibodies to assess the level of nonspecific staining.

### Statistical analysis

Logistic regression was performed to calculate the odds ratio (OR) and 95% confidence interval (CI). In the cell-based cultural model, data present in the study were mean values with standard errors. The significance of differences between samples was analyzed by Student's t test. P<0.05 was considered statistically significant.

## SUPPLEMENTARY MATERIALS FIGURES AND TABLES



## Abbreviations

KS: Kaposi's sarcoma
KSHV: Kaposi's sarcoma-associated herpesvirus
HIV: human immunodeficiency virus
PKC: protein kinase C
NHIRD: Taiwan National Health Insurance Research Database
OR: odds ratio
ESRD: end stage renal disease
HTN: hypertension
CHC: chronic hepatitis and cirrhosis
ORF50p: ORF50 gene promoter
TPA: 12-*O*-tetradecanoyl-phorbol-13-acetate
SB: sodium butyrate
RT-PCR: reverse transcription-PCR
EBV: Epstein-Barr virus
ChIP: chromatin immunoprecipitation
EMSA: electrophoretic mobility shift assay
LANA: viral latency-associated nuclear antigen
