# Supplementary material for: Diabetes and risk of Kaposi's sarcoma: effects of high glucose on reactivation and infection of Kaposi's sarcoma-associated herpesvirus
Source: Oncotarget. 2017 Jul 28;8(46):80595–611. doi: 10.18632/oncotarget.19685 (PMC5655223; doi:10.18632/oncotarget.19685)
Supplement: Supplementary file 1 [file oncotarget-08-80595-s001.pdf]

Diabetes and risk of Kaposi's sarcoma: effects of high glucose on reactivation and infection of Kaposi's sarcoma-associated herpesvirus

SUPPLEMENTARY MATERIALS

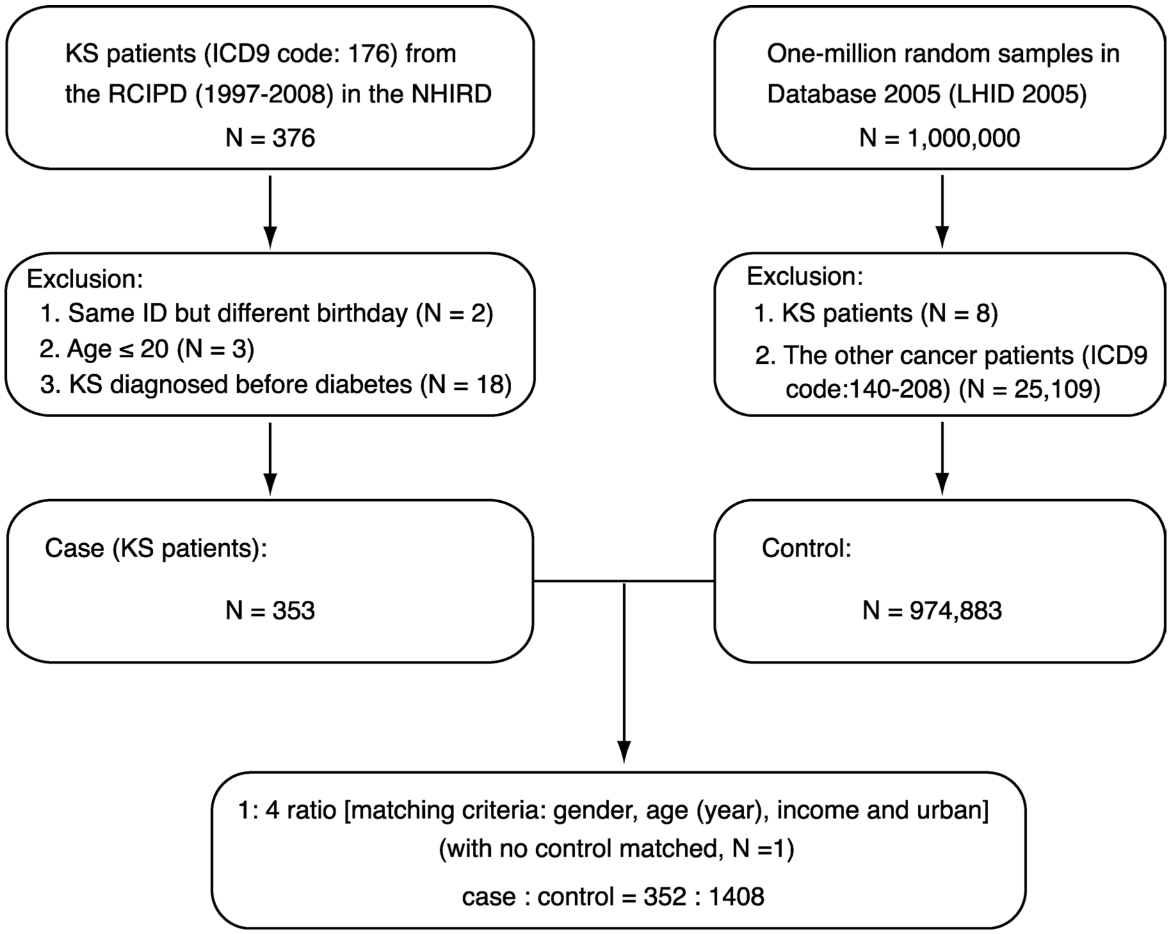

Supplementary Figure 1: The flow chart of sampling procedures for both KS cases and control subjects.

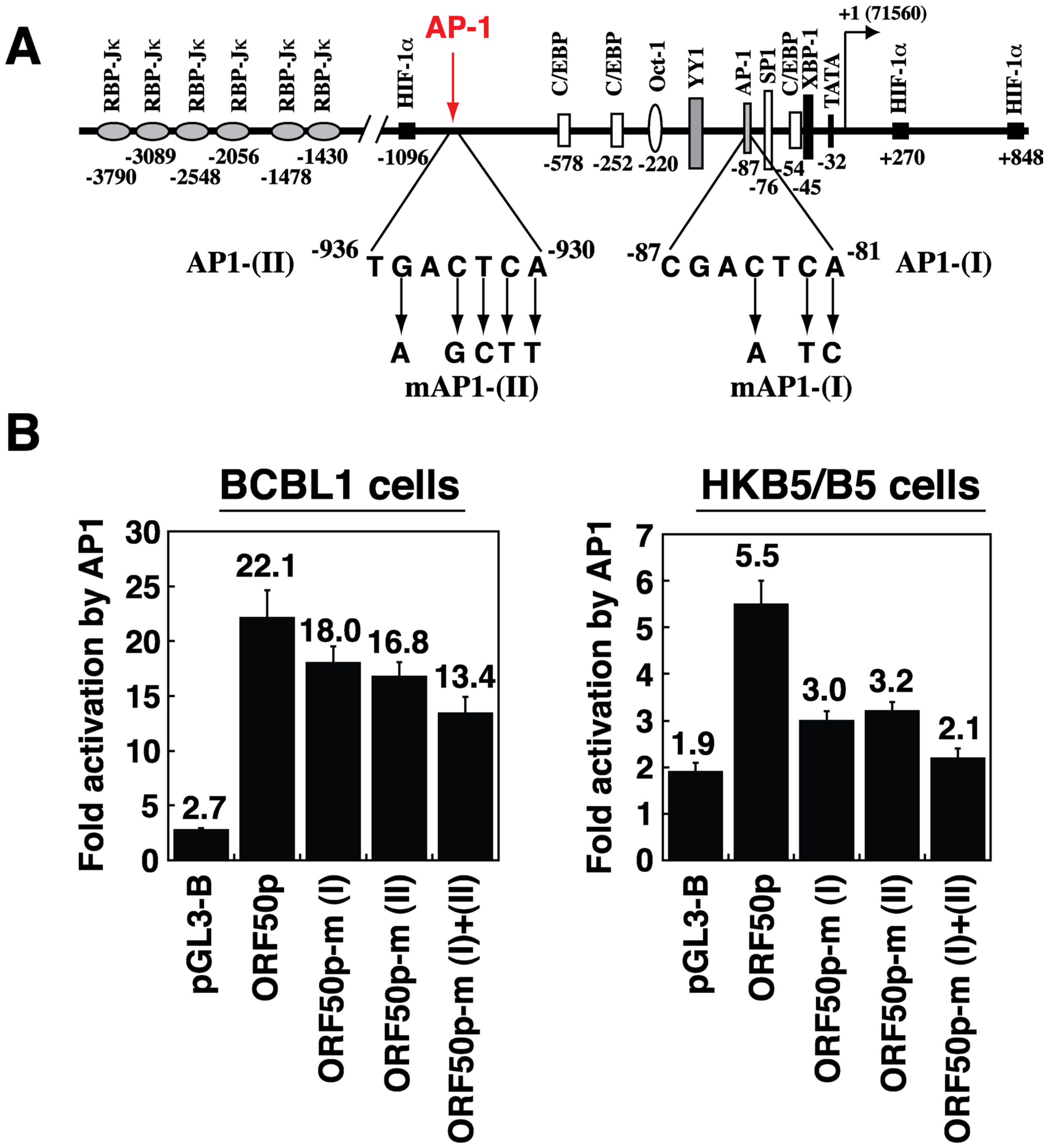

Supplementary Figure 2: Point mutations in the AP1-(I) or AP1-(II) element of the ORF50 promoter impair the promoter activation by AP1. (A) Schematic diagram of the ORF50 promoter and the location of two AP1-binding motifs, AP1-(I) and AP1-(II). **(B)** The luciferase reporter plasmids that contain mutations at the AP1-(I) site, AP1-(II) site, or both AP1 sites were cotransfected with an empty vector or plasmids expressing c-Jun and c-Fos in BCBL1 cells or in HKB5/B5 cells. Forty-eight hours after cotransfection, the response of each reporter construct to AP1 (c-Jun and c-Fos) was determined (n=3).

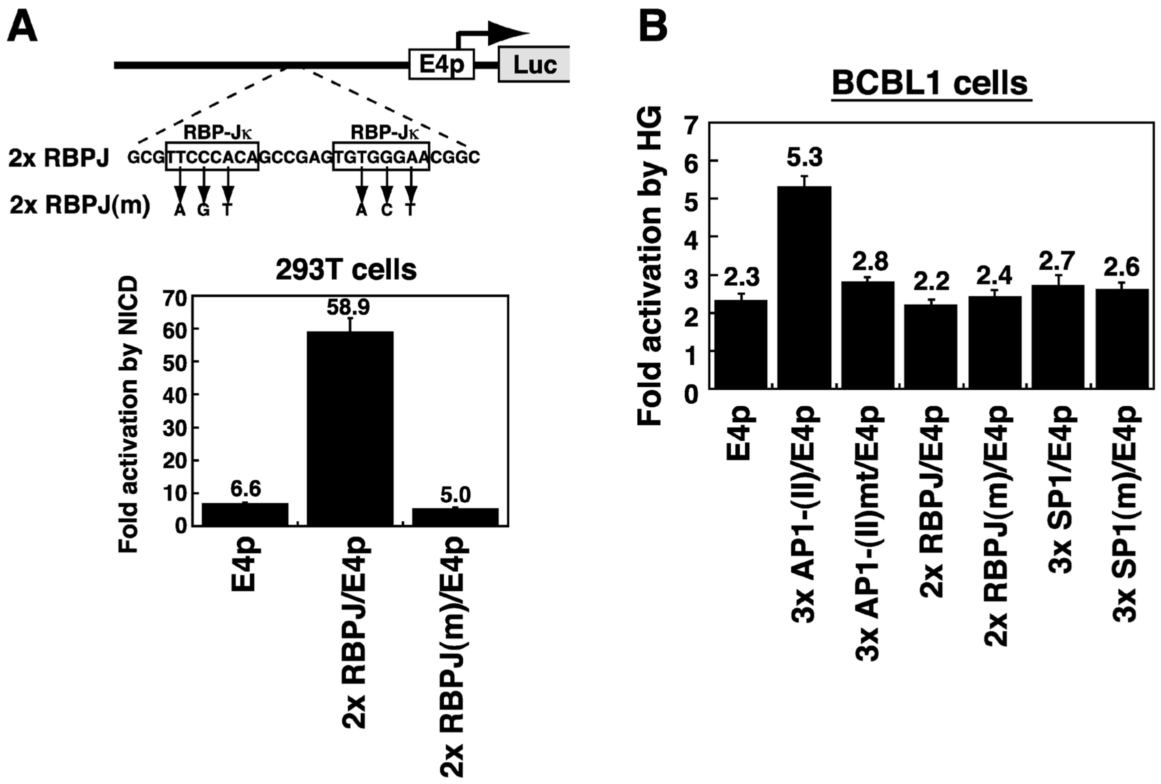

Supplementary Figure 3: The RBP-Jκ- or SP1 element in the ORF50 promoter cannot confer the response to high glucose in BCBL1 cells. **(A)** Activation of the RBP-Jκ-containing reporter plasmid by Notch intracellular domain (NICD) in 293T cells (n=3). **(B)** The luciferase reporter plasmids that contain tandem copies of wild-type or mutated AP1-(II), RBP-Jκ- or SP1 element were transfected into BCBL1 cells, and then the transfected cells were cultured in normal or high glucose for 48 hours. The response of each reporter construct to high glucose was determined by luciferase assays (n=3).

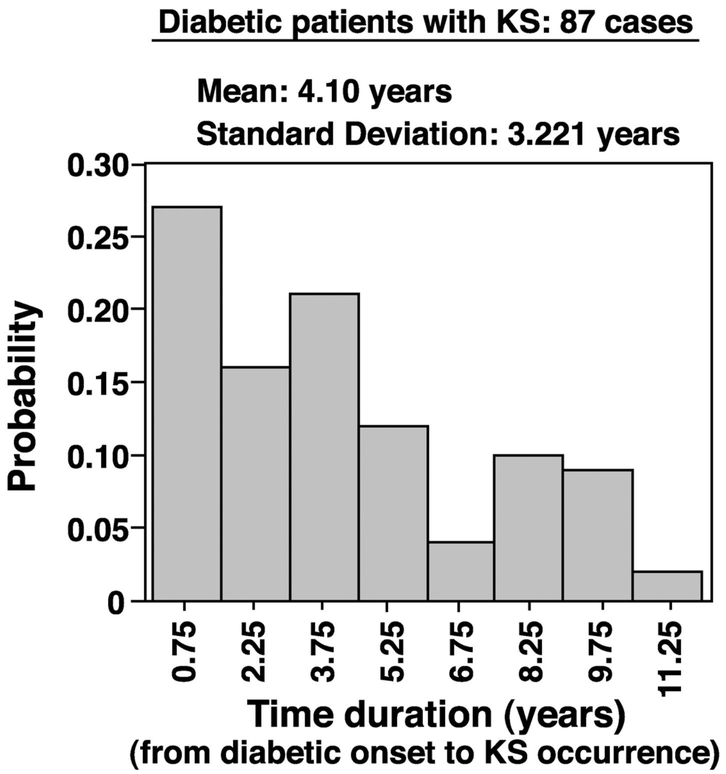

Supplementary Figure 4: Distributions of the time intervals between diabetic onset and KS occurrence in diabetes-associated KS patients. The mean duration of the time intervals from the clinical diagnosis of diabetes to KS occurrence in diabetes-associated KS patients (87 cases) is 4.1 years with a standard deviation of 3.221 years.

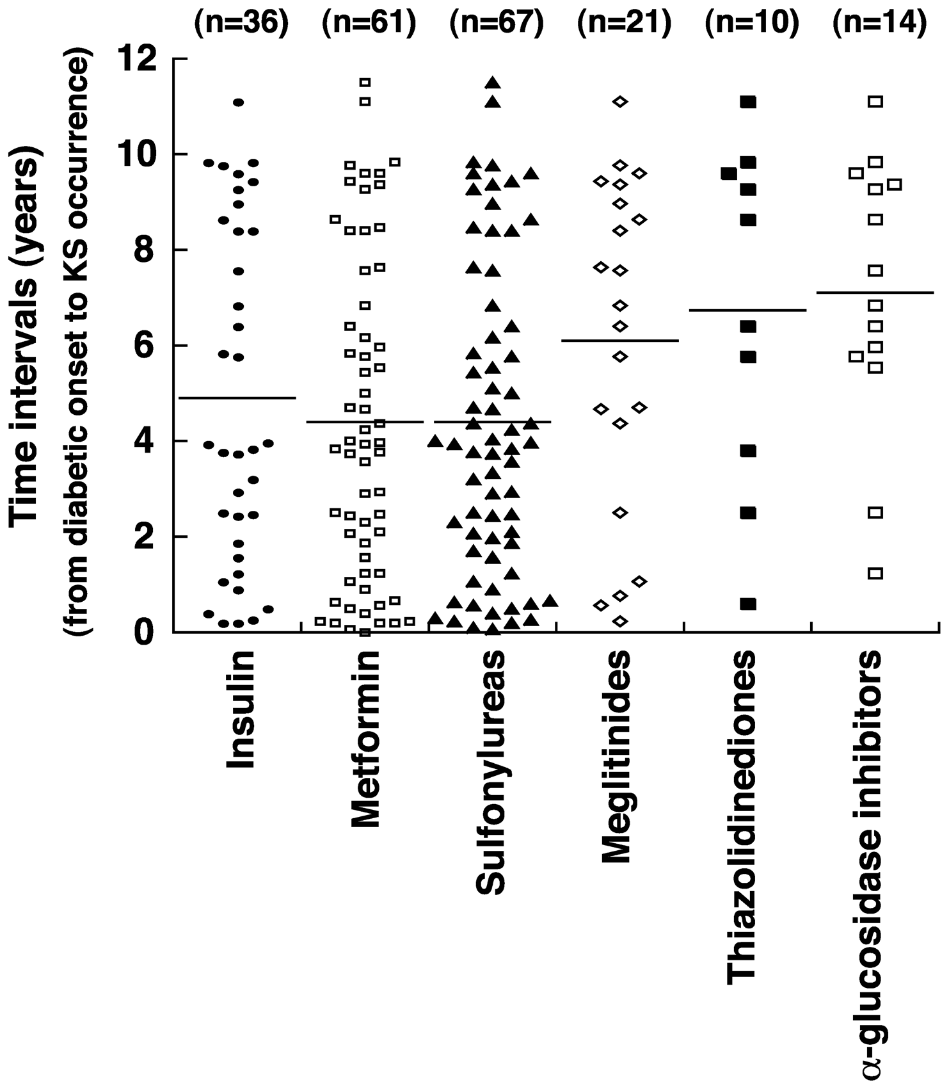

Supplementary Figure 5: Relationship between type of anti-diabetes therapy and KS onset in diabetic patients with KS. A total of 87 diabetes-associated KS patients who had ever used the indicated anti-diabetes drugs (including insulin, metformin, sulfonylureas, meglitinides, thiazolidinediones and alpha-gluosidase inhibitors) were grouped. As noted, most diabetics took more than one anti-diabetes drug. The time intervals between the clinical diagnosis of diabetes and KS were compared among the groups.

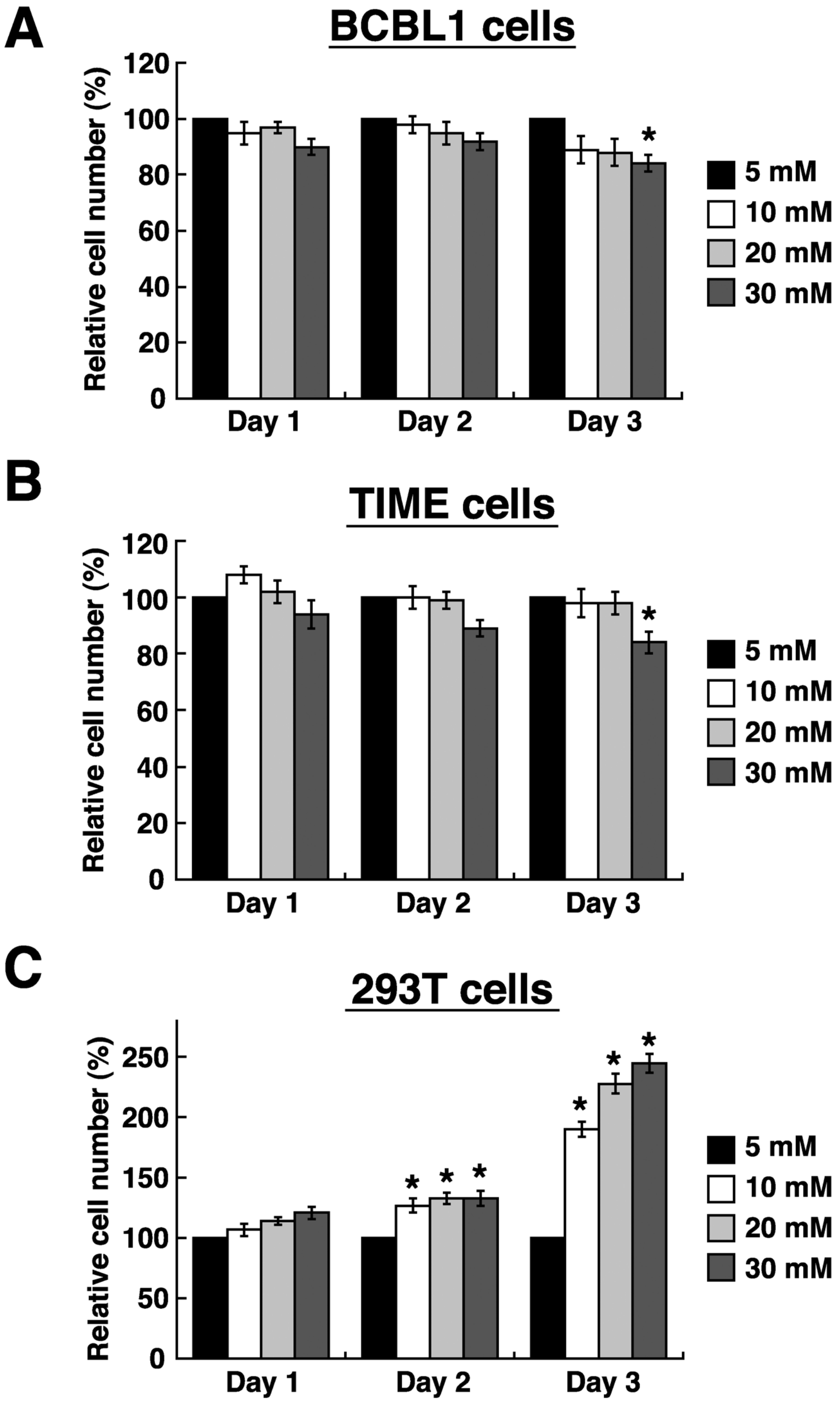

Supplementary Figure 6: Proliferation of BCBL1, TIME and 293T cells under different glucose conditions. Relative cell numbers of BCBL1, TIME or 293T cells cultured in different glucose concentrations were measured at different time points by WST-1 assay. Symbol * indicates significant difference vs. the normal glucose treatment ($P < 0.05$).

Supplementary Table 1: Pattern of anti-diabetes drugs used in the KS group and in the non-KS group

Variables	KS cases (N=87)		Controls (N=193)		p value
	n*	%	n*	%	
DM medication					
Insulin	36	41.38	60	31.09	0.0932
Metformin	61	70.11	136	70.47	0.9525
Sulfonylureas	67	77.01	150	77.72	0.8954
Meglitinides	21	24.14	33	17.10	0.1671
Thiazolidinediones	10	11.49	36	18.65	0.1346
Alpha-glucosidase inhibitors	14	16.09	35	18.13	0.6772
Dipeptidyl petidase 4 (DPP-4)	0	0	0	0	

* Most diabetics took more than one anti-diabetes drug.
